# Quantitative Evaluation of Pectoral Muscle Visualisation as an Indicator of Positioning Quality in Screening Mammography

**DOI:** 10.3390/diagnostics16081218

**Published:** 2026-04-19

**Authors:** Maja Karić, Doris Šegota Ritoša, Petra Valković Zujić

**Affiliations:** 1 Department of Diagnostic and Interventional Radiology, Clinical Hospital Center Rijeka, Krešimirova 42, 51000 Rijeka, Croatia; maja.karic@fzsri.uniri.hr (M.K.); petra.valkovic.zujic@uniri.hr (P.V.Z.); 2 Department of Radiological Technology, Faculty of Health Studies, University of Rijeka, Ul. Viktora Cara Emina 5, 51000 Rijeka, Croatia; 3 Department of Medical Physics and Radiation Protection, Clinical Hospital Center Rijeka, Krešimirova 42, 51000 Rijeka, Croatia; 4 Department for Medical Physics and Biophysics, Faculty of Medicine Rijeka, University of Rijeka, Braće Branchetta 20, 51000 Rijeka, Croatia; 5 Department of Radiology, Faculty of Medicine, University of Rijeka, Braće Branchetta 20, 51000 Rijeka, Croatia

**Keywords:** breast positioning, compression force, mammography, pectoral muscle

## Abstract

**Background/Objectives:** Image quality of mammograms in breast cancer screening is strongly operator-dependent, particularly in the mediolateral oblique (MLO) projection where adequate visualisation of the pectoralis major muscle serves as a surrogate marker of posterior tissue inclusion. Current positioning assessment is predominantly qualitative and subject to inter-observer variability. This study aimed to quantitatively evaluate pectoral muscle visualisation and compression force variability among radiographers participating in a national screening programme. **Methods:** A retrospective observational study was conducted at Clinical Hospital Center Rijeka in January and February 2020. A total of 464 digital MLO mammograms were analysed. Images from nine radiographers were randomly retrieved from the institutional Picture Archiving and Communication System (PACS). Pectoral muscle length and width were measured using a standard clinical workstation with an integrated distance measurement tool. Additional variables included radiographer gender, breast side (LMLO vs. RMLO), imaging order, and applied compression force. Statistical analyses included Welch’s ANOVA, one-way ANOVA, *t*-tests, and appropriate post hoc comparisons. **Results:** Across all MLO projections, the combined mean pectoral muscle width was 41.0 ± 11.4 mm and the mean length was 134.3 ± 21.7 mm. Significant inter-operator differences were observed in pectoral muscle width (*p* < 0.001) and length (*p* = 0.023). Mean muscle width ranged from 35.0 mm to 54.2 mm, and mean length from 126.5 mm to 139.4 mm across radiographers. No significant differences were found with respect to radiographer gender, breast side, or imaging order (all *p* > 0.05). Compression force differed significantly among radiographers (*p* < 0.001), ranging from 117.0 ± 18.3 N to 184.8 ± 33.9 N. **Conclusions:** This study demonstrates significant inter-operator variability in both pectoral muscle visualisation and applied compression force during MLO mammography. These findings indicate that important technical aspects of mammographic examination remain strongly operator-dependent and highlight the need for more consistent positioning practices within screening programmes. Quantitative measurement of pectoral muscle dimensions may serve as a practical and objective approach for monitoring positioning quality and supporting quality assurance in routine clinical practice.

## 1. Introduction

Mammography remains the cornerstone of population-based breast cancer screening, owing to its standardised acquisition protocols, widespread accessibility, and established efficacy in identifying clinically significant lesions at their earliest, most treatable stage [1]. Early detection is paramount; when diagnosed promptly, breast cancer is potentially curable in approximately 90% of cases, significantly reducing the necessity for aggressive treatment and enhancing overall survival outcomes [2]. However, the success of screening programmes is not solely contingent upon advanced in imaging technology—such as full-field digital mammography (FFDM) or digital breast tomosynthesis (DBT)—but is critically dependent on the technical precision and reproducibility of image acquisition [3,4]. Suboptimal positioning or inadequate compression can lead to insufficient tissue inclusion, diminished diagnostic sensitivity, and an increased rate of technical repeats, ultimately impacting both clinical efficacy and resource utilization.

The quality of mammography is inherently operator-dependent, requiring a synergy coordination between the technical proficiency of radiographers in image acquisition, the diagnostic expertise of radiologists in interpretation, and the rigorous oversight of medical physicists in ensuring compliance with quality assurance (QA) protocols [5]. To standardize these processes, the American College of Radiology (ACR), in collaboration with the Centers for Disease Control and Prevention (CDC), has established comprehensive guidelines across all three professions [6]. Similarly, European QA frameworks emphasise robust clinical governance and systematic quality control (QC) procedures [7]. However, a significant discrepancy remains; while physics-based parameters (such as tube voltage or dose) are quantitatively defined and monitored, the adequacy of breast positioning is still largely evaluated using qualitative or semi-quantitative criteria, leading to inherent subjectivity in quality assessment.

**Figure 1:**
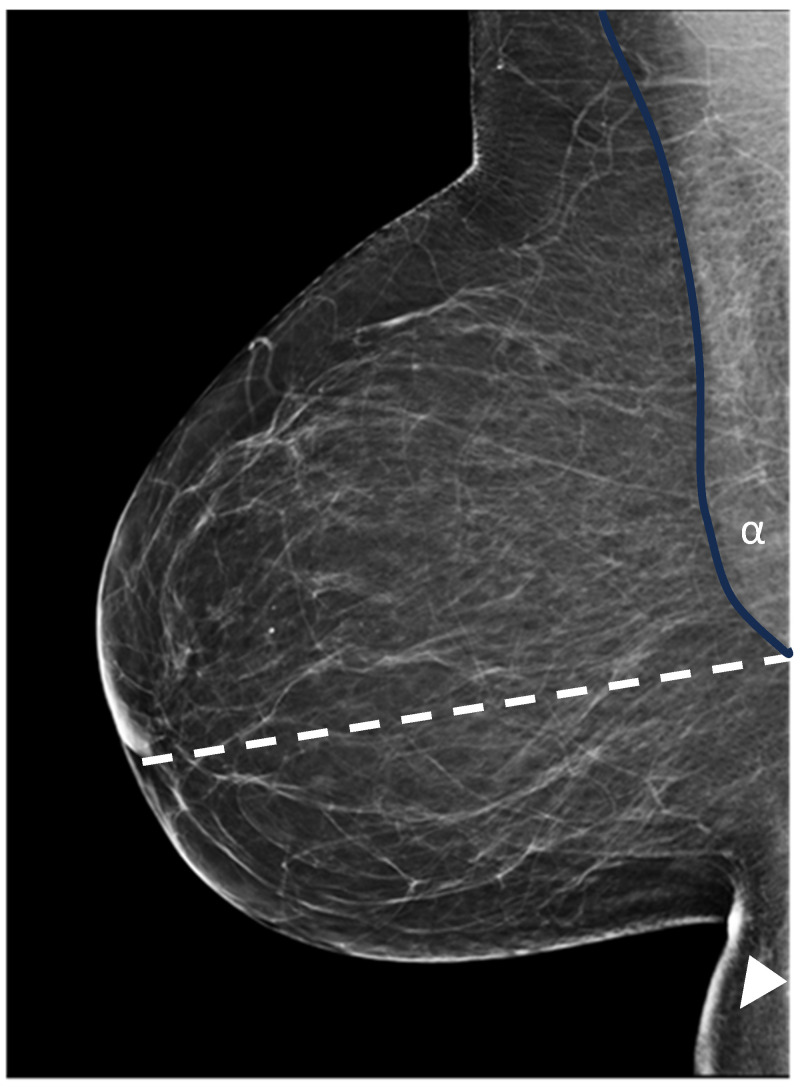
Mediolateral oblique (MLO) projection of the right breast. The posterior nipple line (PNL) is indicated by the dotted line. The pectoralis muscle (should extend to the level of the nipple) should be relaxed (blue line) with an angle > 10° (α). The inframammary fold (arrow head) must be clearly visualized, without overlapping skin folds or air gaps. The nipple must be seen in a profile, without rotation.

Within the standard two-view mammographic protocol (comprising craniocaudal, CC, and mediolateral oblique, MLO projections), the MLO view is paramount for the visualization of posterior breast tissue, specifically the retromammary space and the axillary tail [8,9]. Due to its complex geometric configuration, the MLO projection is highly susceptible to positioning inaccuracies. Even subtle deviations in patient rotation, detector height, or gantry angulation can lead to the exclusion of deep posterior tissue or compromised image interpretability [10,11]. While structured educational programs have been shown to enhance technical proficiency [12], there remains a critical need for objective and reproducible metrics to ensure consistent positioning quality. Visualisation of the pectoralis major muscle on the MLO projection remains a cornerstone for assessing posterior tissue inclusion and the overall technical quality of breast positioning. In routine clinical practice, positioning adequacy is typically verified through the posterior nipple line (PNL) and qualitative evaluation of the muscle’s extension relative to the nipple level [12] (Figure 1). Nevertheless, these established criteria are inherently descriptive, leading to significant inter-observer variability and a lack of objective, reproducible standards for quality control [13,14].

To address this limitation, several studies have proposed quantitative measurements, most notably pectoral muscle length and width—as reproducible indicators of positioning quality [14,15]. Evidence suggests that these dimensions are not necessarily correlated, implying that their independent evaluation may yield complementary information regarding tissue inclusion [16]. Moreover, pectoral muscle width can be influenced not only by patient anatomy but also by operator positioning technique, such as detector angulation and axillary mobilization, making it a complementary metric to muscle length for assessing positioning quality [14]. Furthermore, predictive statistical models based on these measurements have demonstrated the feasibility of estimating compliance with PNL criteria, thereby supporting the translation toward quantitative and partially automated quality control frameworks. Moreover, technical factors, including detector angulation and positioning geometry, significantly influence measured muscle dimensions, suggesting that quantitative parameters reflect a complex interplay between operator performance and acquisition configuration [17]. Recent advances in deep learning and dedicated software have further enabled the automated assessment of breast positioning. Such technologies provide real-time feedback to radiographers, offering a promising solution to further mitigate inter-operator variability and enhance the standardisation of screening programmes [18,19].

Breast mobilisation and compression are also integral components of MLO acquisition. Adequate compression reduces tissue overlap, improves image quality, and decreases the mean glandular dose for the patient [8,20]. It further facilitates appropriate opening of the inframammary fold, which serves as an additional quality indicator in structured audits. Despite the potential for patient discomfort, optimal compression remains essential for ensuring diagnostic reliability [21].

Within the framework of this study, quantitative analysis of pectoral muscle measurements may serve as a robust tool for benchmarking radiographer performance, identifying specific sources of variability, and guiding targeted training. Such interventions are vital for improving positioning technique in screening environments, where reproducibility and standardisation are critical for clinical efficacy [12,22,23,24].

To our knowledge, no previous study has systematically combined quantitative pectoral muscle measurements within a cohort of patients participating in a national screening programme. The aim of this study was to quantitatively evaluate mammographic image quality based on pectoral muscle visualisation among radiographers at Clinical Hospital Center Rijeka involved in the national breast cancer screening programme and to identify opportunities for technique optimisation based on the findings.

## 2. Materials and Methods

### 2.1. Data Acquisition

A retrospective observational study was conducted to evaluate the quality of mediolateral oblique (MLO) mammographic projections based on pectoralis major visualisation among radiographers participating in a national breast cancer screening programme. Prior to the period of image acquisition, all radiographers involved in the screening programme had successfully completed in-house dedicated educational course on mammographic positioning, with particular emphasis on optimal MLO positioning and pectoralis major visualisation. To ensure a standardized evaluation of radiographer performance and to minimize confounding anatomical variables, specific inclusion and exclusion criteria were applied during the retrospective data collection process.

### 2.2. Inclusion Criteria

Inclusion criteria were as follows: asymptomatic women aged 50–69 years participating in the national breast cancer screening programme at the Clinical Hospital Centre Rijeka; availability of a complete bilateral standard mammographic exam consisting of both craniocaudal (CC) and mediolateral oblique (MLO) projections; and images of adequate technical quality for quantitative analysis.

### 2.3. Exclusion Criteria

Exclusion criteria were as follows: previous breast surgery (patients with a history of major breast surgery, including unilateral or bilateral mastectomy or extensive lumpectomy, to avoid bias related to altered breast volume or architectural distortion); breast implants; patients with anatomical deformities (e.g., pectus excavatum or pectus carinatum; patients with significant scoliosis), as these conditions inherently restrict optimal positioning and muscle projection regardless of radiographic technique and symptomatic patients (e.g., presenting with palpable masses, skin thickening, or nipple discharge) who were not suitable for the standardized screening cohort.

### 2.4. Measurement Protocol

The MLO images were acquired and analysed in January and February 2020 at the Department of Diagnostic and Interventional Radiology, Clinical Hospital Center Rijeka. Although the analysis was conducted at that time, the results are presented here for the first time. Importantly, the mammography technique, positioning methodology, and imaging equipment used at the institution have remained unchanged since the time of data collection, ensuring the continued methodological relevance and comparability of the findings. Mammographic images were retrospectively retrieved from the institutional Picture Archiving and Communication System (PACS) for women who underwent standard 2D mammography as part of the national screening programme. For nine radiographers, images were randomly selected, and the order of breast acquisition (left-first vs. right-first) was recorded to assess potential systematic differences in positioning quality. Eligible images were technically adequate MLO projections suitable for pectoralis major measurement, while images in which the muscle could not be reliably delineated due to incomplete posterior depiction, pronounced artefacts, or technical issues were excluded. This approach ensured that measured variability reflected radiographer performance rather than limitations in image quality.

For each image, two quantitative parameters of pectoral muscle depiction were recorded: length and width following similar methodology as described by Bentley et al. [15]. Measurements were performed on digital images using a standard clinical workstation with an integrated distance measurement tool, preserving the original image geometry.

**Figure 2:**
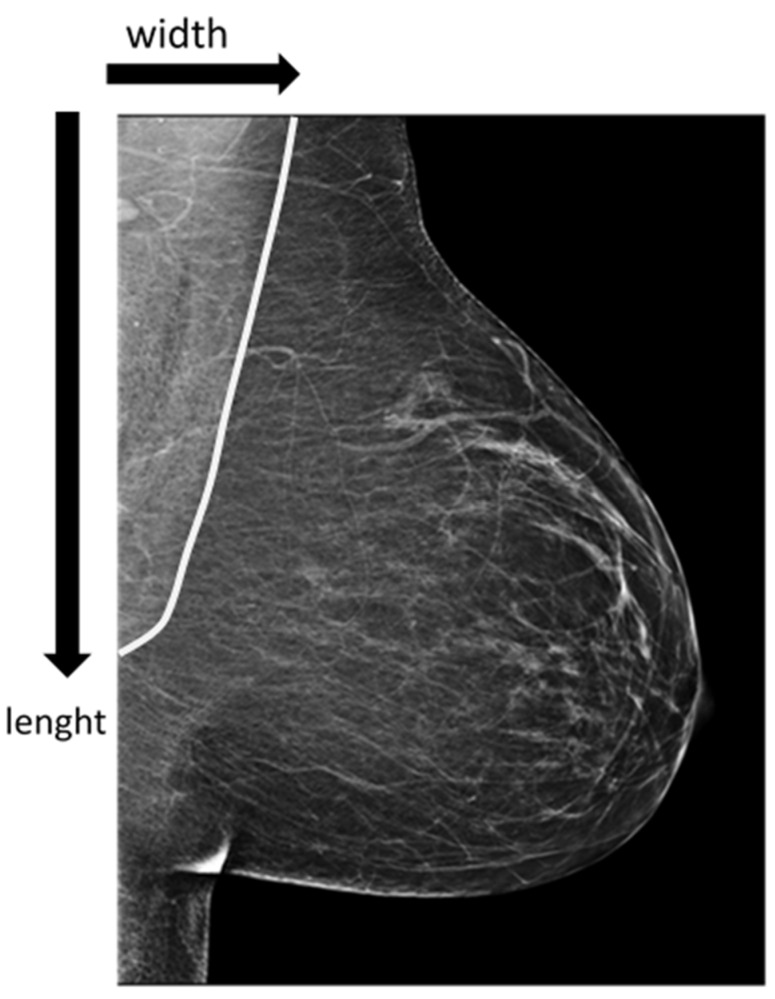
Quantitative assessment of the pectoralis major on MLO projections was performed using standardized linear metrics. The anterior boundary of the muscle was identified by the distinct radiopaque margin of the muscular shadow (marked by the white line). Muscle length was defined as the maximum longitudinal extension measured along the principal oblique axis from the superior margin to the inferior most visible point. Muscle width was recorded as the maximum transverse thickness, measured perpendicular to the principal axis within the superior segment of the muscle.

All measurements were performed by a single trained investigator to ensure internal consistency. The investigator was blinded to radiographer identity, as all images were anonymized except for a non-identifiable radiographer code. Although a standardized measurement protocol based on established literature was used to minimize variability, no formal intra- or inter-observer reproducibility assessment was conducted, as reproducibility analysis was not the primary aim of this study. To minimise variability, a standardised viewing protocol with consistent display format and magnification was applied. Muscle width was defined as the maximum transverse thickness perpendicular to this axis in the superior segment. Muscle length was defined as the maximum extension along the principal oblique axis, parallel to the dominant posterior border. Muscle width was measured as the maximum transverse thickness at the most prominent point. In cases ofirregular or indistinct borders, measurements were standardized by usingthe most consistently visible segment across all images (Figure 2). Additional recorded variables included radiographer identifier, radiographer gender, side of acquisition (L-MLO or R-MLO), imaging order, and applied compression force.

Mean pectoral muscle dimensions were first calculated across all combined MLO projections, followed by calculation of mean dimensions for each radiographer. These values were then compared across the nine radiographers and analyzed for associations with radiographer gender, side of acquisition (L-MLO vs. R-MLO), imaging order (left first vs. right first), and applied compression force.

### 2.5. Statistical Analysis

All statistical analyses were performed using TIBCO Statistica (versions 13.x for analyses in 2020 and 14.3.0 for recent analyses; TIBCO Software Inc., Palo Alto, CA, USA) and Python (versions 3.8 and 3.14; Python Software Foundation, Wilmington, DE, USA) with the statsmodels, SciPy, and scikit-posthocs libraries. A significance level of *p* = 0.05 was used.

- Data normality was assessed using the Kolmogorov–Smirnov test.
- Continuous variables were summarized as mean ± standard deviation (SD).
- Homogeneity of variances was evaluated using Levene’s test.
- For comparisons among more than two groups, classical one-way ANOVA was applied when Levene’s test confirmed homogeneity of variances. Welch’s ANOVA was used when variances were unequal. Post hoc pairwise comparisons were performed using Tukey’s test after one-way ANOVA or Games–Howell test after Welch’s ANOVA, with Holm adjustment for multiple comparisons.
- Independent samples *t*-tests were used for comparisons between two groups when normality and homogeneity assumptions were met; these included comparisons of pectoral muscle dimensions and compression force between radiographer gender or breast sides (LMLO vs. RMLO).
- Relationships between compression force and pectoral muscle dimensions were assessed using Spearman’s rank correlation due to non-normal distribution of compression force, while pectoral muscle width and length were approximately normally distributed. Correlation coefficients (r) and *p*-values are reported for each comparison.

## 3. Results

A total of 464 digital MLO images were retrospectively collected, comprising 52 images for each of eight radiographers and 48 images for one radiographer. The cohort included four female and five male radiographers. As the standard screening protocol involves bilateral imaging, the dataset predominantly consisted of paired MLO projections (left and right breasts).

**Figure 3:**
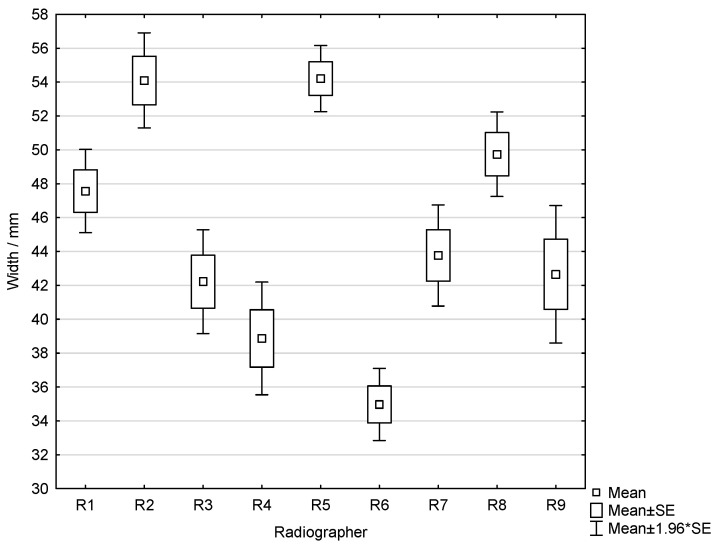
Box-and-whisker plot showing the distribution of pectoral muscle width among radiographers.

Across all MLO projections, the combined mean pectoral muscle width was 41.0 ± 11.4 mm, with a range of 7.8–73.0 mm. The mean length was 134.3 ± 21.7 mm, ranging from 49.1 mm to 191.2 mm. The result of Welch’s ANOVA revealed a statistically significant difference in mean pectoral muscle width among radiographers (*p* < 0.001). Mean pectoral muscle widths ranged from 35.0 mm (R6) to 54.2 mm (R2), indicating substantial variability in muscle visualization across operators (Figure 3).

**Figure 4:**
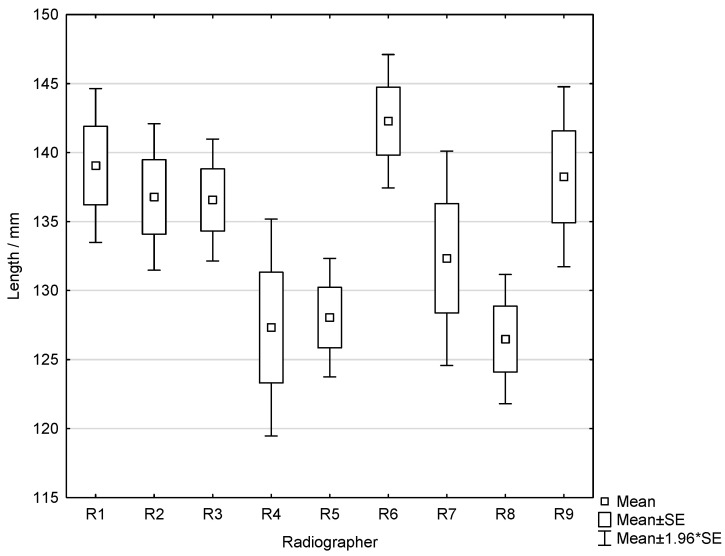
Box-and-whisker plot showing the distribution of pectoral muscle length among radiographers.

Welch ANOVA also showed a significant difference between radiographers in pectoral muscle length (*p* = 0.023). Mean lengths ranged approximately from 126.5 mm to 139.4 mm across radiographers (Figure 4).

**Figure 5:**
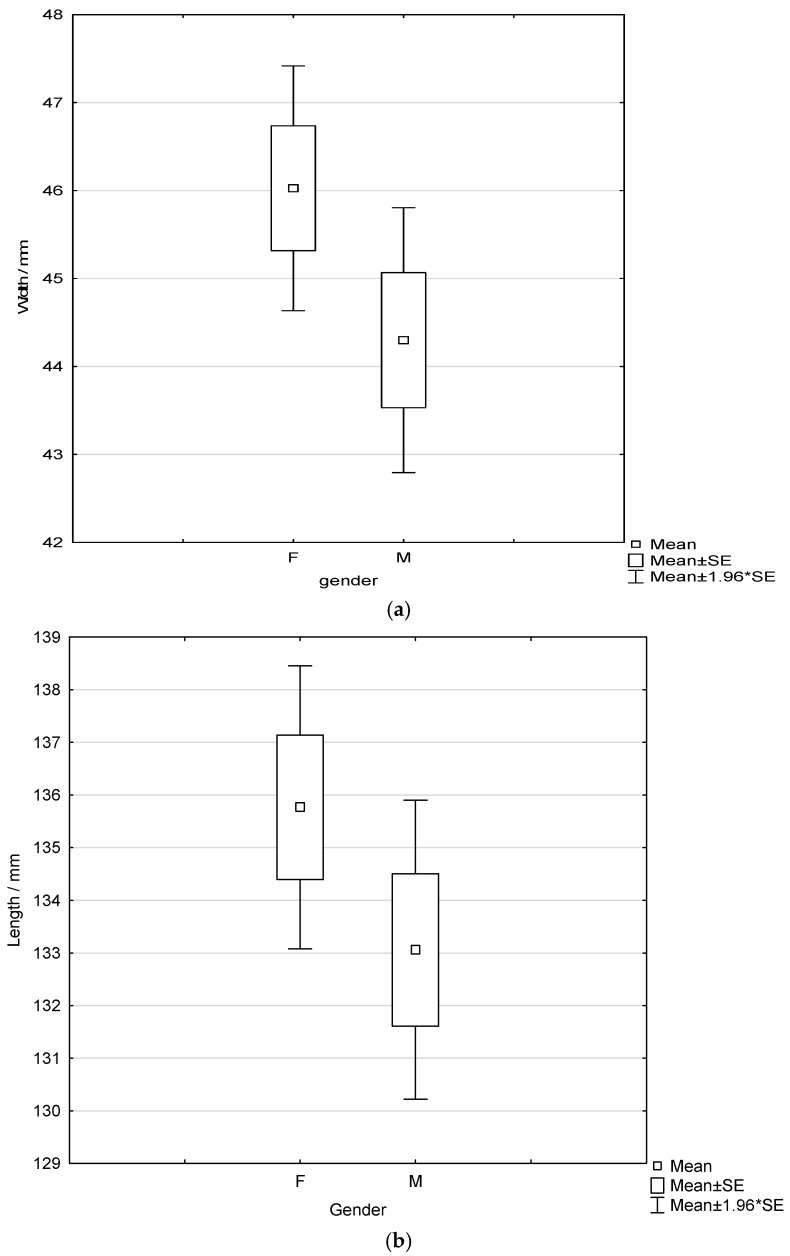
Box-and-whisker plots showing the width (**a**) and length (**b**) of the pectoral muscle for female and male radiographers.

Analysis of the differences in pectoral muscle width and length between female and male radiographers using independent samples *t*-tests showed no statistically significant differences for either variable (width: *p* = 0.105; length: *p* = 0.182) even though values were higher for female radiographers (Figure 5).

**Figure 6:**
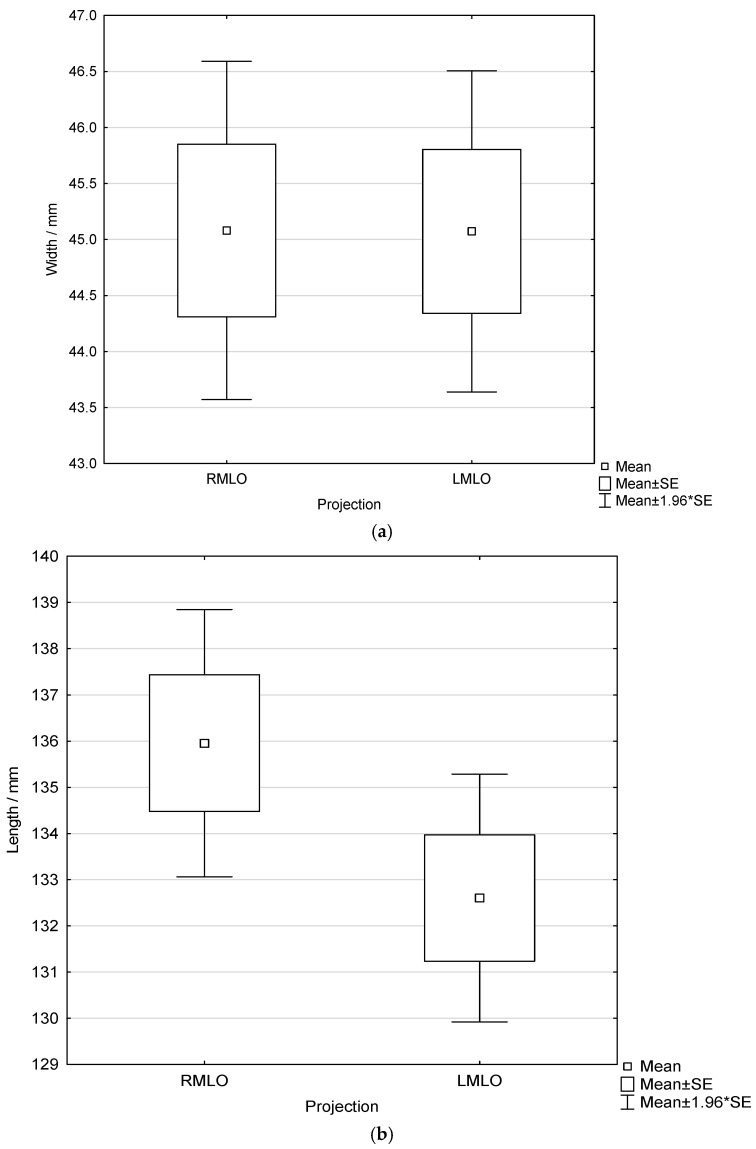
Box-and-whisker plots showing the width (**a**) and length (**b**) of the pectoral muscle in LMLO and RMLO views.

No statistically significant differences were found in width or length between the LMLO and RMLO views, as assessed by independent samples *t*-tests (*p* = 0.994 and *p* = 0.097, respectfully) (Figure 6).

**Figure 7:**
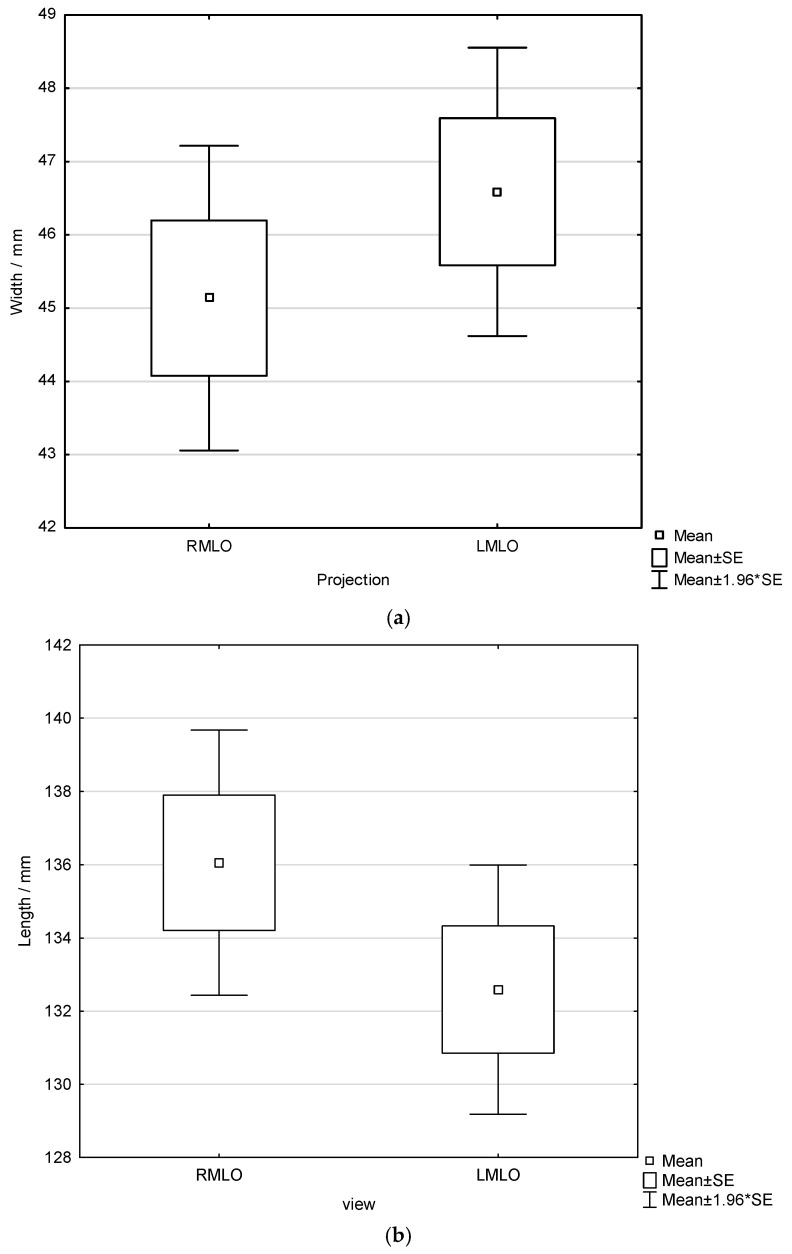
Width (**a**) and length (**b**) of the pectoral muscle in LMLO and RMLO projections when left breast is imaged first.

**Figure 8:**
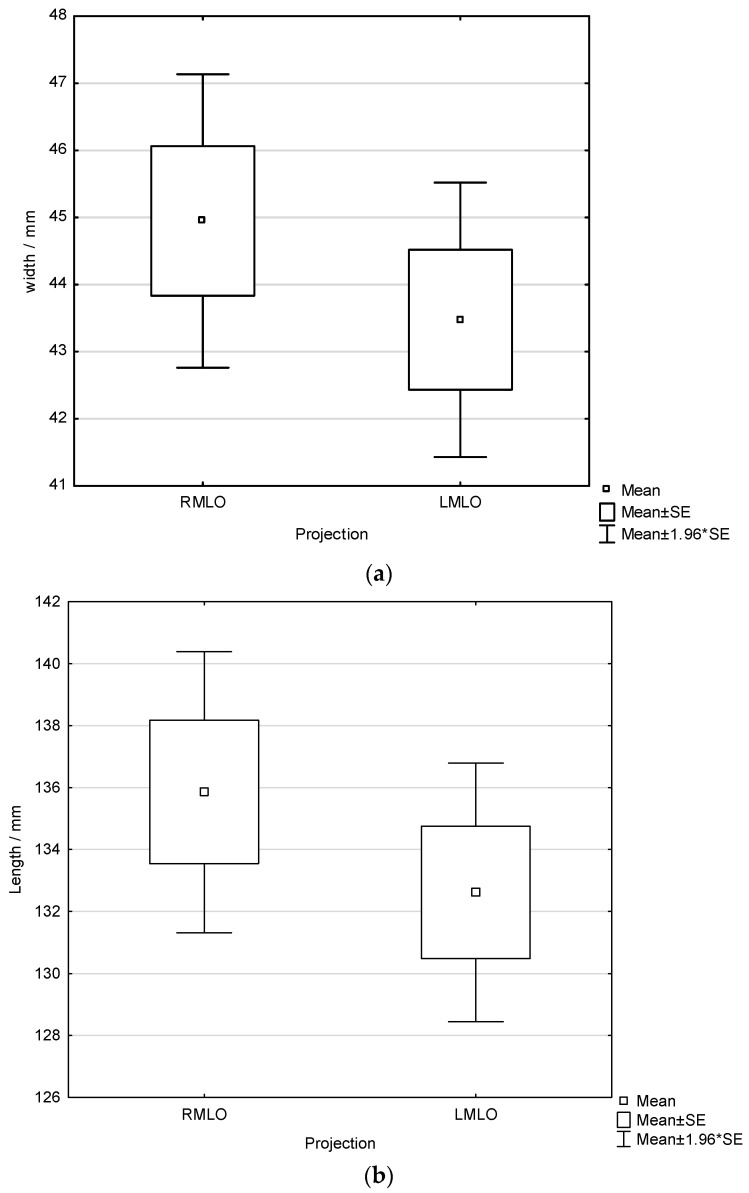
Width (**a**) and length (**b**) of the pectoral muscle in LMLO and RMLO projections when right breast is imaged first.

Furthermore, pectoral muscle width and length on RMLO and LMLO projections were analyzed with respect to the order of breast imaging (Figure 7 and Figure 8). A non-significant trend toward greater muscle width on the side imaged first was observed (one-way ANOVA across all radiographers: *p* = 0.321 for left-first and *p* = 0.337 for right-first imaging). Pectoral muscle length was likewise not significantly influenced by imaging order (independent samples *t*-test: *p* = 0.173 for left-first and *p* = 0.304 for right-first imaging). Additionally, subgroup analysis using independent samples *t*-test was performed for each radiographer to assess whether pectoral muscle width differed between RMLO and LMLO views according to imaging order. Only one radiographer (R8) demonstrated a statistically significant difference in both pectoral muscle width and length, and only when the left breast was imaged first (*p* = 0.028 for width and *p* = 0.044 for length). No statistically significant differences in pectoral muscle width or length related to imaging order were observed for the remaining radiographers.

**Figure 9:**
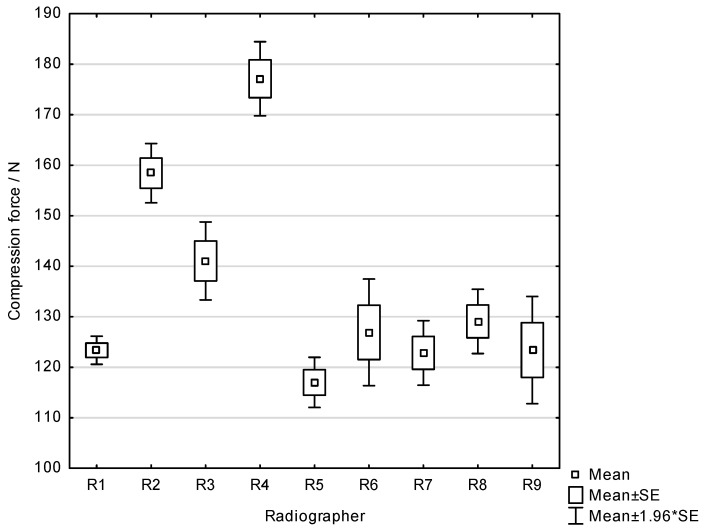
Compression force applied for MLO projections for all Radiographers (R1–R9).

Welch’s one-way ANOVA demonstrated a statistically significant difference in compression force among radiographers (*p* < 0.001), indicating variability in applied compression force across operators. The highest mean compression force was recorded for Radiographer 4 (184.8 ± 33.9 N), followed by Radiographer 2 (158.0 ± 21.56 N). The lowest mean compression force was observed for Radiographer 5 (117.0 ± 18.3 N) (Figure 9). Games–Howell post hoc analysis revealed that Radiographer 4 applied a significantly higher compression force compared with all other radiographers (all *p* < 0.001). Radiographer 2 also applied significantly higher compression force compared with several other radiographers. In contrast, no statistically significant differences were found among radiographers with lower mean compression forces (R1, R5, R6, R7, R8, and R9). Compression force below 100 N was observed in 33% of the images. The coefficient of variation (CV) ranged from 8.3% to 31.7% across radiographers. Analysis of the coefficient of variation for pectoral muscle width and length revealed that radiographers exhibiting the highest intra-operator variability in compression force did not necessarily show the highest variability in positioning metrics.

Spearman correlation analysis was performed to explore the relationship between applied compression force and pectoral muscle dimensions. No statistically significant correlations were observed (force vs. width: r = −0.09, *p* = 0.0504; force vs. length: r = −0.059, *p* = 0.1999), suggesting that variations in compression force do not directly explain the observed differences in muscle visualization.

## 4. Discussion

This study quantitatively evaluated mammographic positioning quality using pectoral muscle dimensions on MLO projections in patients participating in a national screening programme. The results demonstrated statistically significant inter-operator variability in both pectoral muscle width and length, as well as in compression force applied. In contrast, no significant differences were observed with respect to radiographer gender, breast side (LMLO vs. RMLO), or imaging order. Since the acquisition protocol and equipment configuration have remained unchanged, the findings remain methodologically relevant despite the retrospective nature of the dataset.

### 4.1. Pectoral Muscle Dimensions and Inter-Operator Variability

The pectoral muscle dimensions measured in this study were compared with previously published data. The overall pectoral muscle width was 41 ± 11.4 mm and the mean length was 134.3 ± 21.7 mm on MLO projections. Comparable measurements have been reported in the literature. Bentley et al. [15] described a mean pectoral muscle width of 48 mm (range 18–150 mm) and a mean length of 139 mm (range 75–200 mm). Similarly, Spuur et al. [14] reported a mean width of 46.5 mm (range 10–86 mm) and a mean length of 140.2 mm (range 33–218 mm). In comparison with these studies, the mean pectoral muscle length observed in the present study is closely aligned with previously reported values, while the mean width is slightly lower. However, both measurements fall within the ranges described in the literature. These findings suggest that pectoral muscle inclusion on MLO projections in the present study is comparable to that reported in previous studies.

The observed significant differences in pectoral muscle width and length among radiographers indicate that positioning technique remains an important source of variability, even within a structured national screening programme. Because all images were acquired under the same institutional protocol, the observed variability most likely reflects differences in individual positioning practice rather than equipment-related factors. Pectoral muscle visualisation on the MLO view is widely accepted as a surrogate marker of posterior tissue inclusion. Greater muscle extension generally reflects improved mobilisation of posterior breast tissue and adequate detector angulation. However, muscle dimensions are influenced not only by operator technique but also by patient anatomy and positioning geometry [15,25,26]. Therefore, observed variability among radiographers may reflect not only differences in individual technique but also patient-level factors such as breast volume, tissue composition, and mobility, which were not controlled in this study. As a result, the present findings support the concept that quantitative muscle measurements can serve as reproducible technical indicators rather than absolute measures of quality.

Importantly, muscle width and length were not uniformly distributed across radiographers, suggesting that these two parameters may capture different aspects of positioning performance. This aligns with previous observations that length and width are not necessarily strongly correlated and may provide complementary information. Incorporating both parameters into quality audits could therefore improve the sensitivity of performance monitoring.

Although an in-house training session was conducted prior to data collection, the observed variability highlights the need for targeted refresher training focused specifically on MLO positioning to enhance pectoral muscle visualisation and posterior tissue inclusion. Such focused instruction could help reduce inter-operator differences and further improve image quality in national screening practice

### 4.2. Gender, Side, and Imaging Order

No statistically significant differences were found between female and male radiographers. Although slightly higher mean width and length of the pectoral muscle were observed among female radiographers, these differences were not significant. This suggests that technical performance is not inherently gender-dependent but rather related to training, experience, and individual technique.

Similarly, no systematic differences were found between LMLO and RMLO views or according to imaging order. The absence of consistent first-side effects indicates that fatigue or workflow sequencing did not meaningfully influence positioning quality at the group level. The isolated significant finding in one radiographer likely reflects individual technique variability rather than a systematic effect.

### 4.3. Compression Force Variability

Beyond the variability observed in pectoral muscle dimensions, the present study demonstrated substantial inter-operator variability in applied compression force during mammographic examination. A statistically significant and large difference was observed between radiographers indicating that compression force is strongly dependent on the individual radiographer rather than being uniformly applied across practitioners. Radiographer 4 applied the highest mean compression force (184.78 ± 33.85 N), which was significantly greater than that of all other radiographers. The magnitude of this difference was clinically notable, with an average difference of approximately 60 N compared to several colleagues. Radiographer 2 also applied relatively high compression force (158.44 N ± 21.56) and differed significantly from multiple radiographers. Compression force below 100 N was observed in 33% of the images, indicating that a substantial proportion of examinations were performed with relatively low compression, which may have implications for image quality and absorbed dose delivered to the patient. Although our study did not directly assess the relationship between compression force, image quality, and absorbed dose, previous studies had shown that increased compression force is associated with improved mammographic image quality and decreased breast thickness, which in turn can reduce mean glandular dose, supporting the clinical relevance of compression force as a technical parameter in mammography optimization [27,28,29]. The observed weak correlations between compression force and pectoral muscle dimensions suggest that variations in applied compression do not directly account for the differences in muscle visualization, highlighting the predominant role of individual positioning technique. The CV of compression force reflected considerable intra-operator variability. The higher CV values suggest inconsistent application of compression force rather than systematic control, indicating variability in technique. Wade et al. [30] showed also large variability between radiographers (95–143 N) within one breast centre. Also, Ramnarain et al. [31], Branderhost et al. [32], and Mercer et al. [33] in their research regarding compression force also reported variations between radiographers and not units.

There are currently no evidence-based recommendations regarding optimal breast compression in mammography. The European guidelines for quality assurance in breast cancer screening and diagnosis state that “the breast should be properly compressed, but no more than is necessary to achieve a good image quality” [3]. The guidelines from the National Health Service Breast Screening Programme in the UK state that “the force of the compression on the x-ray machine should not exceed 200 Newtons or 20 kg” [34]. The lack of precise and objective recommendations for breast compression might lead to variations in applied compression between radiographers and breast centres [35].

The primary aim of this part of this study was to reduce variability in the compression force applied by radiographers and emphasise the importance of avoiding substandard compression forces, whether they are too high or too low. Many factors contribute to the variability in the compression force, namely the client’s breast volume and compressibility, their tolerance to pain, the equipment’s compression paddle and use of automated compression force, and the radiographer [30]. Adequate breast compression is a fundamental component of mammographic image acquisition. It reduces breast thickness, improves tissue separation, decreases motion artefacts, enhances image quality, and contributes to mean glandular dose reduction [3,7,8]. At the same time, excessive compression is associated with increased pain and discomfort, which may negatively affect patient satisfaction and adherence to breast screening programmes [10,36,37]. Fear of pain has been identified as a potential barrier to participation in mammography screening [11]. Therefore, the marked inter-operator variability observed in the present study may have implications not only for technical image quality but also for patient experience and screening compliance, as the substantial differences between radiographers suggest a lack of standardization in compression technique. Overall, these findings highlight the need for greater consistency in compression practice, as reducing operator-dependent variability may improve the uniformity of image quality, optimize mean glandular dose, and enhance patient comfort, ultimately contributing to improved quality assurance in mammography [38].

Pectoral muscle visualisation and compression force are widely recognised surrogate indicators of positioning adequacy and image acquisition technique. In this context, the observed inter-operator differences in pectoral muscle dimensions, together with variability in compression force, suggest inconsistent adherence to positioning and acquisition standards across radiographers. While these findings do not directly demonstrate reduced diagnostic image quality, they indicate that optimal imaging conditions are not consistently achieved, which may result in variability in image adequacy, particularly in terms of posterior tissue inclusion.

### 4.4. Implications for Structured Quality Assurance and AI Integration

From a quality assurance perspective, the present results support the integration of quantitative positioning metrics into routine audits. Traditional assessment of MLO adequacy relies largely on qualitative evaluation of the posterior nipple line and muscle depiction, which is subject to inter-observer variability. Quantitative measurement of pectoral muscle dimensions offers objectivity, reproducibility, and the possibility of identifying outliers and monitoring longitudinal performance trends.

While the present study did not directly investigate AI-assisted positioning assessment, the quantitative metrics evaluated here—pectoral muscle width and length—could potentially serve as input features or validation parameters in automated systems. Previous research has demonstrated that deep learning–based algorithms can detect suboptimal positioning patterns and quantify technical parameters associated with image adequacy [18,37]. These observations highlight a future perspective in which AI tools may provide real-time feedback to radiographers, enhance consistency, reduce repeat examinations, and support continuous professional development. Practical implementation and clinical validation of such systems remain to be investigated in future studies.

### 4.5. Limitations

This study has several limitations that should be considered when interpreting the findings. First, intrinsic patient-related factors, such as breast volume, anatomical variation, and tissue composition were not controlled. These variables significantly influence both the mechanical requirements for compression and the achievable dimensions of the pectoralis major muscle on the MLO projection. As this was a retrospective study conducted in a high-volume clinical environment, volumetric and anthropometric data were not collected as part of this retrospective dataset,

However, we mitigated this potential bias through the random retrieval of images from the PACS system and the inclusion of a substantial sample size, ensuring that a diverse range of breast morphologies was presented across all radiographers. Second, all pectoral muscle measurements were performed manually by a single trained investigator using a standardized clinical workstation. The investigator was blinded to radiographer identity, as images were anonymized except for a non-identifiable radiographer code. Although a strictly standardized measurement protocol based on established literature was applied to minimize variability, no formal intra- or inter-observer reproducibility assessment was conducted. Future studies should include multi-reader reproducibility testing and comparison with automated, deep-learning-based positioning assessment tools. Furthermore, we acknowledge the need to bridge the gap between observed variability and clinical quality. In the context of a national screening programme, standardisation is a fundamental prerequisite for quality. Our findings demonstrate that even among trained professionals, significant differences persist in compression force and muscle visualization. This variability indicates that the “optimal” possible image is not being consistently achieved across all operators. By definition, a high degree of operator dependency results in inconsistent posterior tissue inclusion and non-standardized breast compression, which are recognised determinants of image acquisition adequacy. However, as diagnostic image quality and clinical outcomes were not directly assessed in this study, these findings should be interpreted as indicators of potential variability in image adequacy rather than direct evidence of reduced diagnostic performance.

Third, the exclusion images may have introduced a selection bias by excluding the most poorly positioned exams. Consequently, the observed variability likely represents a “best-case” assessment of a routine clinical work.

Fourth, measurement reproducibility was not formally assessed, and LMLO and RMLO images from the same patient were treated as independent, which may influence the robustness of the reported variability estimates.

Finally, while this study establishes technical variability, it does not directly correlate these metrics with clinical endpoints such as cancer detection rates or specific recall outcomes. Therefore, the results should be interpreted within the framework of technical quality assessment rather than direct clinical performance evaluation. In addition, although 33% of images had compression force below 100 N, the relationship between lower compression force and pectoral muscle visualization was not directly assessed and warrants investigation in future studies to better define optimal compression thresholds for image quality. Future prospective studies are warranted to explore the direct associations between quantitative positioning metrics, diagnostic efficacy, and patient-reported experience measures (PREMs) to further validate these parameters as longitudinal quality indicators in national screening programmes.

## 5. Conclusions

This study demonstrates significant inter-operator variability in both pectoral muscle visualisation and applied compression force during MLO mammography within a national screening programme. Quantitative assessment of muscle width and length provides an objective method for evaluating positioning performance and identifying opportunities for technical optimisation. The observed variability in compression practice further highlights the need for greater standardisation, as reducing operator-dependent differences may improve the uniformity of image acquisition, optimise mean glandular dose, and enhance patient comfort.

Integration of structured quantitative monitoring with AI-assisted positioning assessment may represent an important step toward more objective, reproducible, and patient-centered and more standardized practice in breast cancer screening programmes.

In response to these findings, structured protocols and targeted training and education sections have been proposed within our department to reduce inter-operator variability and promote harmonisation of mammographic technique.

## Data Availability

The original contributions presented in this study are included in the article. Further inquiries can be directed to the corresponding author.
